# Predicting future fallers in Parkinson’s disease using kinematic data over a period of 5 years

**DOI:** 10.1038/s41746-024-01311-5

**Published:** 2024-12-05

**Authors:** Charalampos Sotirakis, Maksymilian A. Brzezicki, Salil Patel, Niall Conway, James J. FitzGerald, Chrystalina A. Antoniades

**Affiliations:** 1 NeuroMetrology Lab, Nuffield Department of Clinical Neurosciences, University of Oxford, Oxford, UK; 2 Nuffield Department of Surgical Sciences, University of Oxford, Oxford, UK

**Keywords:** Neurology, Health care

## Abstract

Parkinson’s disease (PD) increases fall risk, leading to injuries and reduced quality of life. Accurate fall risk assessment is crucial for effective care planning. Traditional assessments are subjective and time-consuming, while recent assessment methods based on wearable sensors have been limited to 1-year follow-ups. This study investigated whether a short sensor-based assessment could predict falls over up to 5 years. Data from 104 people with PD without prior falls were collected using six wearable sensors during a 2-min walk and a 30-s postural sway task. Five machine learning classifiers analysed the data. The Random Forest classifier performed best, achieving 78% accuracy (AUC = 0.85) at 60 months. Most models showed excellent performance at 24 months (AUC > 0.90, accuracy 84–92%). Walking and postural variability measures were key predictors. Adding clinicodemographic data, particularly age, improved model performance. Wearable sensors combined with machine learning can effectively predict fall risk, enhancing PD management and prevention strategies.

## Introduction

Falls are a common problem for people living with Parkinson’s disease (PD)^1–4^, with consequences including injuries, hospitalisations, and reductions in mobility, quality of life, and life expectancy^5–8^. A recent review estimated that some 60% of all people living with PD have experienced at least one fall^9^. People with PD who fall report a greater fear of falling, impairment of balance, freezing and motor fluctuations than their less frequently falling peers^10–16^.

Several risk factors for falling have been identified^17^. A history of having already fallen is the most obvious predictor of future falls^9^. Gait slowing, increased walking variability^18^ and postural instability^19,20^, are associated with increased probability of falling. At present, evaluating these usually relies on the subjective judgement of the clinician^21,22^. A more objective way to prospectively identify future fallers before their first fall is desirable.

Wearable sensors are an increasingly popular way of identifying movement problems in people with PD^23^. Data from an array of wearable sensors analysed using automatic feature selection strategies can distinguish between people with PD and other parkinsonian disorders^24^, track the progression of motor symptoms in PD and other parkinsonian disorders^25–27^, and identify motor symptoms such as tremor and freezing of gait^28^. As summarized in Table 1, recent research has advanced fall risk estimation in people with PD by integrating digital monitoring systems with clinical assessments. People with PD who have been clinically phenotyped as postural instability and gait disturbance (PIGD) are indeed more prone to falls^29^. Wearable sensors can, therefore, enhance fall risk estimation by analysing walking features^30,31^. In addition, there seems to be a strong association between walking variability and increased risk of falls^32^, which highlights the importance of continuous digital measurements over subjective clinical observations. Weiss et al.^32^ showed that using a single lumbar accelerometer worn for 3 days can prospectively identify future fallers within a year. However, this remote passive monitoring paradigm may be logistically challenging for many clinical setups. Whether a shorter, standardised, clinic-based gait and balance assessment can be utilised to predict future fallers remains unknown.

**Table 1:** Summary of literature on predicting future falls in Parkinson’s disease using digital devices

Study	Aim	Task	Results	Follow-up [months]
*Digital device* *+* *clinical assessment*				
Pelicioni et al. (2019)^29^	Contrast fall rates between PIGD and non-PIGD PD subtypes. Clinical assessment is used to classify phenotypes.	113 PD (67 PIGD, 46 non-PIGD) Prospective study; recording of gait-related features using triaxial accelerometer; also measured other disease and clinically relevant features.	PIGD more likely to fall overall with more falls related to FOG, balance-related falls, and at home.	12
Shah et al. (2023)^70^	To investigate if digital measures of gait collected passively over a week of daily activities in people with PD increases the discriminative ability to predict future falls compared to fall history alone. Mixed group of fallers and non-fallers at time of recruitment.	34 PD (17 fallers, 17 non-fallers). 3 IMU sensors for one week of passive gait monitoring. Followed up by email every 2 weeks for a year for self-reported falls.	Inertial sensors worn on the feet and lumbar level for 7 days provided measures of gait pace, variability and turning that increased the ability to predict future falls in addition to history of previous falls	12
Ullrich et al. (2023)^71^	To compare different data aggregation approaches and machine learning models for the prospective prediction of fall risk using gait parameters derived either from continuous real-world recordings or from unsupervised gait tests. (FallRiskPD study). Mixed fallers and non-fallers.	35 PD patients with foot-worn sensors performing unsupervised 4 x 10 m walking tests over two weeks. Falls were self-reported for 3 months.	The highest accuracy (74%) was achieved with a Random Forest Classifier applied to the passive monitoring gait data, when aggregating all walking bouts and days of each participant	3
Tsai et al. (2023)^47^	To evaluate the feasibility of combining disease-specific and balance-related measures as risk predictors for future falls in patients with PD. Mixed fallers and non-fallers.	95 patients underwent postural sway measurements and clinical functional scores. Followed up patients to determine if fall occurred after 6 months.	Fall history, Tinetti balance, sway length, velocity, and gait score associated with future falls.	6
*Digital devices only*				
Weiss et al. (2014)^32^	Whether metrics derived from three-day continuous recordings were associated with fall risk. Non-fallers followed for one year to evaluate predictors of transition from non-faller to faller.	67 non-falling PD patients wore an accelerometer on lower back for 3 days.	Higher than median gait variability was associated with higher risk of progressing to fall in the follow-up period	12
Ma et al. (2022)^72^	To evaluate gait features associated with higher risk of falls. Mixed fallers and non-fallers (both groups had history of falls)	51 PD patients assessed using six wearable gyroscopes and accelerometers during 7 m timed up and go task	Increased gait variability was associated with increased risk of falls	6
Sturchio et al (2021)^73^	Whether kinematic features can predict increased risk of falls. Most participants already falling at the time of recruitment.	26 PD patients with orthostatic hypotension assessed using two-minute walk test, timed up and go and postural sway tasks	Waist sway, jerkiness, centroidal frequency predicted increased risk of falls	6
Greene et al. (2021)^74^	Whether the number of falls can be estimated from kinematic parameters. Mixed datasets of falling and non-falling participants were used.	71 patients with PD compared with healthy older population. Participants were assessed using a three-metre timed up and go test with two sensors placed on shins	There was a moderate correlation between sensor data and actual fall counts	Up to 6

*PIGD* postural instability and gait disturbance phenotype, *FOG* freezing of gait, *IMU* inertial measurement unit.

The aim of the present study was to determine whether data acquired by wearable sensors during a brief test in clinic can objectively predict the risk of falling in people with PD. We used data from an ongoing longitudinal PD cohort study, Oxford Quantification in Parkinsonism (OxQUIP). We utilised machine learning methods to analyse baseline wearable sensor measurements taken from participants at their first study visit, together with follow up data at 24, and 60 months that specified whether the participant had had any falls by each time point. Additionally, in order to deliver clinically interpretable model results we explored the most important kinematic features for the identification of potential fallers.

## Results

Using IMU data from baseline, we aimed to predict future faller status at 24 and 60-months’ time horizons using classification algorithms.

One hundred and four participants were eligible for inclusion in the analysis. Demographic and clinical information are shown in Table 2. Thirteen participants had fallen by 24 months, and 23 by the 60-month follow-up.

**Table 2:** Summary of demographics for 60-month time window

Parameter median (standard deviation)	Fallers	Non fallers
*N*	23	74
Age (IQR)	71 (6.5)	65 (9.8)
Sex (male/female)	14/9	42/32
Months since diagnosis (IQR)	100.4 (98)	30.4 (47.3)
Side of symptom onset (right/left)	10/13	34/40
Dominant side (right/left/ambidextrous)	22/0/1	61/10/3
Montreal cognitive assessment score (IQR)	27 (3)	27 (3)

*IQR* inter-quartile range.

### Variability of walking most consistently predicts future falls

First, feature selection was performed using the whole dataset of 104 participants at each time point. Differences were tested with Mann–Whitney *U* test and corrected using a 1% false discovery rate (FDR). Supplementary Table 1 highlights those features demonstrating significant differences between fallers and non-fallers at a 60-month horizon. A comprehensive overview of the statistical results is available in Supplementary Table 2. Specifically, the analysis revealed significant differences in features related to walking and postural variability in terms of standard deviation between fallers and non-fallers, in addition to mean stride length and age at enrolment. These included the variability of single and double limb support phases, swing and terminal double support phase durations, as well as the variability of postural sway acceleration. Spearman correlation analysis revealed an expected correlation between swing and stance and at least several other features. The notable non-correlated features were trunk range of motion on the sagittal plane, step duration and toe off angle variability (Supplementary Fig. 1).

### Shorter time horizons yield more reliable predictions in five classifier models

Next, the majority class (non-fallers) was resampled to match the size of the minority class (fallers) to ensure class balance at each time point. We then trained and evaluated 5 machine learning models using the resulting dataset (*n* = 26 at 24 months and *n* = 46 at 60 months).

Evaluation of classification benchmarks for Logistic Regression, Random Forest, Support Vector Machines, Elastic Net and XGBoost is presented in Supplementary Table 3. These models were used as they are known to deal well with collinearities that were expected to be apparent in the datasets. For each model, fivefold cross-validation was performed on the set of features. The dataset was randomly split in 5 sets; 4 sets are used to train the model and the remaining one is used as an independent validation set.

In order to explore the predictive power of a model trained with kinematic data only we compared it to a combined one containing both kinematic and clinicodemographic data. We further tested the model performance before and after the feature selection process. This resulted in four different feature sets at which the model was trained:

a) Complete feature set including clinico-demographics

b) Complete feature set excluding clinico-demographics

c) Selected features (based on univariate testing), including clinico-demographics

d) Selected features excluding clinico-demographics

**Fig. 1:**
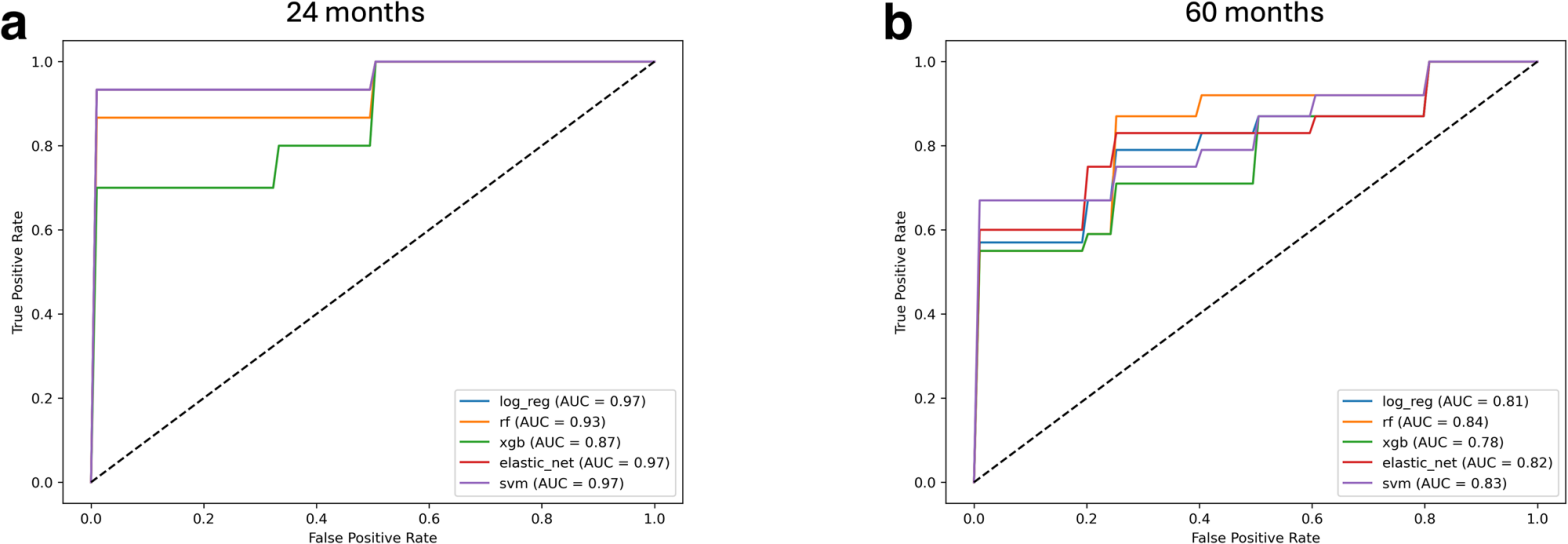
Receiver operating characteristic (ROC) curves. Five classifiers were evaluated in the study: Logistic Regression (log_reg), Random Forest (rf), Support Vector Machine (svm), Elastic Net and XGBoost at each of the **a** 24, and **b** 60 months. The performances of the best models are illustrated and trained on the selected set of features. AUC area under the curve.

Receiver operating characteristic curves (ROC) for the models are presented in Fig. 1. The models revealed excellent performance (AUC > 0.9, accuracy = 84–92%) in the 24-month time and diverged performance in the 60-month time window. For the 60-month time window, RF trained on the selected features, including the clinicodemographic data, performed the best (AUC = 0.85, accuracy = 78%; see Supplementary Table 4). Notably, only the age at enrolment was significant and included in the model and did not substantially improve the classification performance (without age: AUC = 0.81, accuracy = 78%).

### Comparison with clinical scales and risk factors

**Fig. 2:**
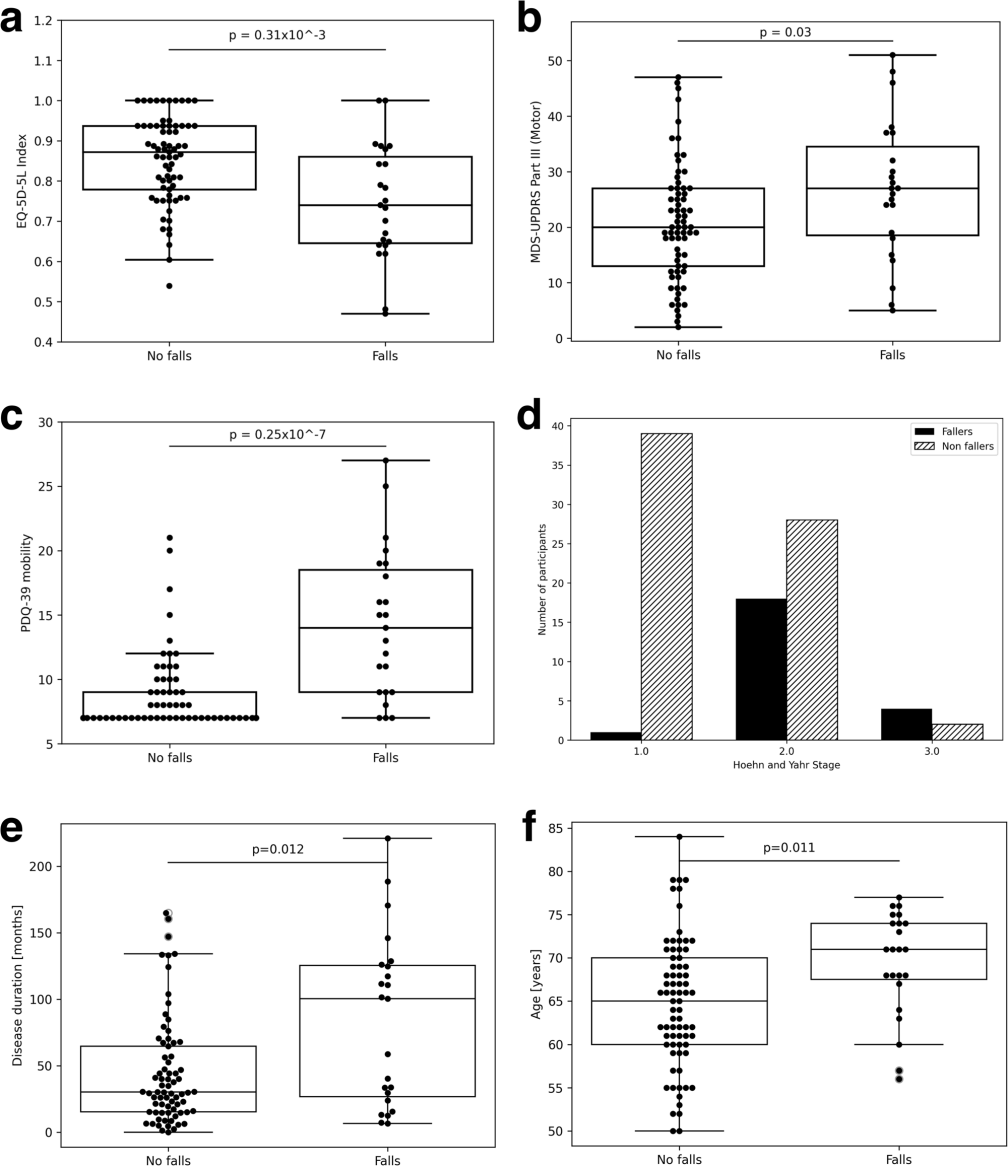
Comparison of clinically assessed risk factors of falls in the studied population. **a** EQ-5D-5L (EuroQol—5 Dimensions—5 Levels) index, **b** Movement disorders society-Unified Parkinson’s disease rating scale-part 3 (MDS-UPDRS-III), **c** PDQ39mobility which is the sum of items 4–10 of PDQ39 questionnaire (self-rated walking, fear of falling, confidence in getting around), **d** number of participants in both groups, per Hoehn and Yahr (H&Y) stage, **e** disease duration and **f** age.

We used several commonly employed questionnaires and clinical scales to capture disease severity and self-perceived decline in mobility. There was a difference between prospective fallers and non-fallers in terms of their baseline Motor (Part III) score of the MDS-UPDRS, Hoehn and Yahr stage, PDQ39 (mobility) and EQ-5D-5L index scores (UK version) (Fig. 2). The baseline PDQ39 mobility items that were significantly worse in the group of participants who started falling included difficulties with walking 100yds, ½ mile, getting around the house, getting around in public, fear of falling, need of assistance when going out, and the associated feeling of being confined to the house.

## Discussion

Our study presents a digital approach to predicting first falls in patients with PD using wearable sensor data. This 5-year prospective study was designed to investigate the predictive capability of kinematic data derived from two standard motor clinical assessments, namely, a 2-min walking task and a 30-s postural sway examination. We assessed five machine learning classifier models for their ability to identify people with PD, with no prior history of falls, who are at risk of falling within 2 and 5 years.

Of the five models assessed, the Random Forest classifier outperformed the other models at 60 months. At 24 months, Logistic Regression, Elastic Net, and Support Vector Machine models were the best performing. All five classifiers were most accurate at 24 months. A decline in accuracy at longer time points is a general feature of forecasting and is, therefore, to be expected to some degree, but it is a reasonable assumption that variability in disease progression rate is a contributory factor here. The kinematic data on which the analysis was based was all derived from a single visit for each participant, and it, therefore, contains no individual information about progression.

Fall risk prediction based on inertial sensor data is common in geriatric populations. Typically, studies aiming to predict older fallers focus on people who are already falling^33^. For instance, Rehman et al.^34^ and Qiu et al.^35^ using walking and mobility tests demonstrated an accuracy of 89% and 98%, respectively in identifying people who have already experienced a fall. Lockhart et al.^36^ and Howcroft et al.^37^ used walking to predict fallers prospectively, as we are doing for PD patients in this study. They demonstrated 82% and 57% accuracy respectively. The lower accuracy compared to Rehman and Qiu studies is to be expected given the prospective study design. The present study model performance scores are comparable to the Lockhart (2021 study).

Other studies have reported different rates of falls in PD^12,20,29,38^. For the non-falling subset of Weiss et al.^32^, 14 out of 67 (20.9%) experienced their first fall in a 12 month follow-up period. In a more recently published extension of the LONG-PD study, by 24 months, 32 out 846 (3.7%) participants were falling “persistently”, which was defined as still experiencing falls in at least 2 annual follow-ups^39^. The discrepancy in falling rates between studies can be explained by differences in disease stage at recruitment, recall bias, and varying proportions of PIGD and tremor-dominant phenotypes. The patients in our study were relatively early in the course of their disease, mostly at Hoehn and Yahr stages 1 or 2, and it is therefore expected that our event indices fall on the lower end of the spectrum seen in the literature. In keeping with other studies, metrics such as baseline Hoehn and Yahr disease stage, MDS-UPDRS Part III, PDQ39 mobility, and general self-reported disability (EQ-5D-5L index) proved to be significantly worse in people who experienced their first fall within the follow-up period of this study.

Feature selection was undertaken to identify the key variables that distinguish people with Parkinson’s with and without risk of falling. Mean stride length was found to be an important predictor of future falls. Notably, all the other important predictors were standard deviations of a range of gait parameters, i.e. measures of variability. The significance of walking variability in relation to falls has been well established in previous research^30,32,40,41^, exploring falling both in healthy old adults and people living with neurological disorders. Accurate measurement of walking variability is a key advantage of wearable systems since such variability is difficult to estimate with the naked eye. The decrease in stride length observed in the participants at risk of falls may suggest a compensatory mechanism to manage deteriorating dynamic balance.

The natural next step, if we are able to predict the risk of falls, is to offer a medical intervention to prevent their occurrence. Several treatment programmes and fall prevention strategies have been proposed^42,43^. These focus on exercise^44–46^, fall-prevention education^47,48^, medication reviews^49–52^, and complex care interventions^53,54^. Clinical trials are currently underway to identify pharmaceutical agents that may prevent falls^55^. Our findings could improve participant selection for these trials so that resources can be focused on the population with a significant risk of falling within the study timeline. Reports show that fall prevention strategies can be successful^56–58^ when offered at an earlier stage, especially before the first fall, but a comprehensive falls prevention programme is resource intensive, and a model that predicts who does or does not need to be offered such a programme could help in population health and social care planning. One of the main focuses of this study was to develop patient-centric diagnostic and prognostic tools. The method used delivers accurate predictions of fall risk, while the brevity of the assessment minimises the burden on people living with PD.

The major limitation of the study is its reliance on participant recall of fall occurrence as the ground truth for the classifier algorithms. In the same vein, while telephone follow-ups offer a convenient method for reporting falls and can facilitate broader participation, they may lack the precision of in-person visits. Recall bias could potentially affect the reliability of the ground truth for falls. We attempted to mitigate this limitation in several ways. First, we derived fall metrics from history taking obtained by a trained clinician, as well as through self-reported questionnaires and recorded clinical communications. Whenever possible, we sought confirmation of the fall, or lack thereof, through collateral history. Finally, times and fall characteristics were confirmed by contemporaneous clinical records whenever possible. In this study, we compared various analysis pipelines with cross-validation, ensuring proper data splitting and processing within each fold to avoid pitfalls such as data leakage. However, to ensure the generalisability of the final result, our classifier model also needs to be validated using a separate test set. Partitioning the dataset in this study in order to hold part of it as a final test set would have rendered the training set very small and the test set inadequate for meaningful model evaluation, and a further validation study will, therefore, be needed to do this. Ideally, this would be done prospectively by an independent group, and as part of routine clinical assessment to evaluate real-life utility. This would investigate whether the accuracies presented in this analysis can be replicated in a more heterogeneous population.

The current study contributes to the understanding of the risk of falls in PD showing that wearable IMU kinematics and machine learning can be used to identify potential fallers in PD from a single gait and posture data collection session lasting less than 3 min. The study presents a comprehensive benchmarking of various models and analysis pipelines. These models were designed based on two primary objectives: First, to identify people with PD at risk of falling. Second, to ensure the reliability of the models by making the data processing methods transparent to clinical experts. Early identification of potential fallers may pave the way for more targeted and effective management strategies of PD, ultimately helping to prevent life-threatening falls.

## Methods

### Participants

**Fig. 3:**
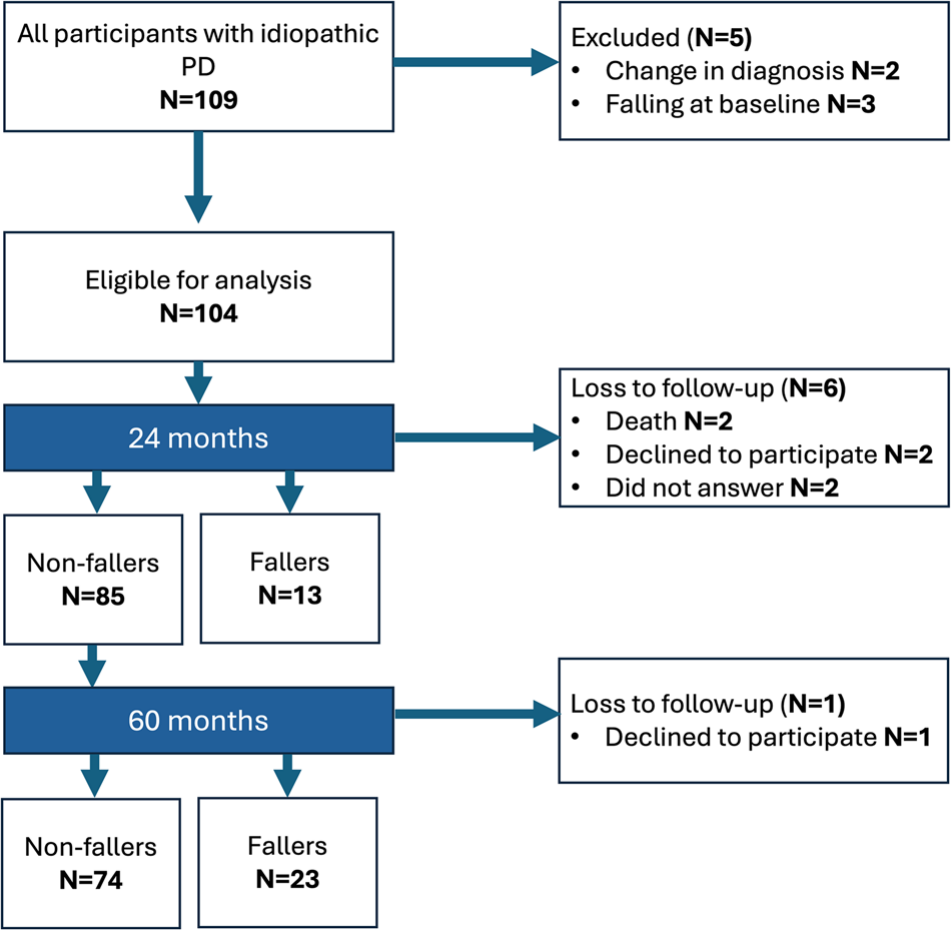
Follow-up flowchart. Data were analysed at two time points: 24 and 60 months. Initially, 109 participants with idiopathic PD were recruited. Participants were lost to follow up leaving 98 participants for the 24-month time period (13 fallers and 85 non-fallers) and 97 participants for the 60 months (23 fallers and 74 non-fallers).

We analysed data from one hundred and nine participants with mild-to-moderate^59^ idiopathic PD who were recruited into the Oxford Quantification in Parkinsonism (OxQUIP) study, a longitudinal cohort study assessing multiple digital biomarkers. Ethical approval was obtained from the University of Oxford under reference number 16/SW/0262). All participants were informed about the study’s aims and protocols and gave their informed written consent to participate in the study and for publication of the study results. The study inclusion criteria were (1) probable idiopathic PD diagnosis given by a movement disorder physician, as per Movement Disorder Society (MDS) clinical diagnosis criteria^60^, (2) age of 50 or older, (3) ability to walk for 2 min and stand still for 30 s and (4) Montreal Cognitive Assessment (MoCA)^61^ score greater than 24. The exclusion criteria were: (1) inability to perform tasks independently (without any assistance or walking device), (2) inability to follow instructions, (3) any other (than PD) neurological or psychiatric disorder. Three participants were excluded because they had already had falls at the time of baseline assessment, and two were excluded because their diagnosis was changed during the study period. This left 104 patients in the study group. Falls status was verified by surveying participants and their families during clinical history taking. A full flowchart of participant selection and follow-up is available in Fig. 3. Participants lost to follow-up were not considered in the fall prediction modelling at 60 months.

### Baseline gait and sway tasks

**Fig. 4:**
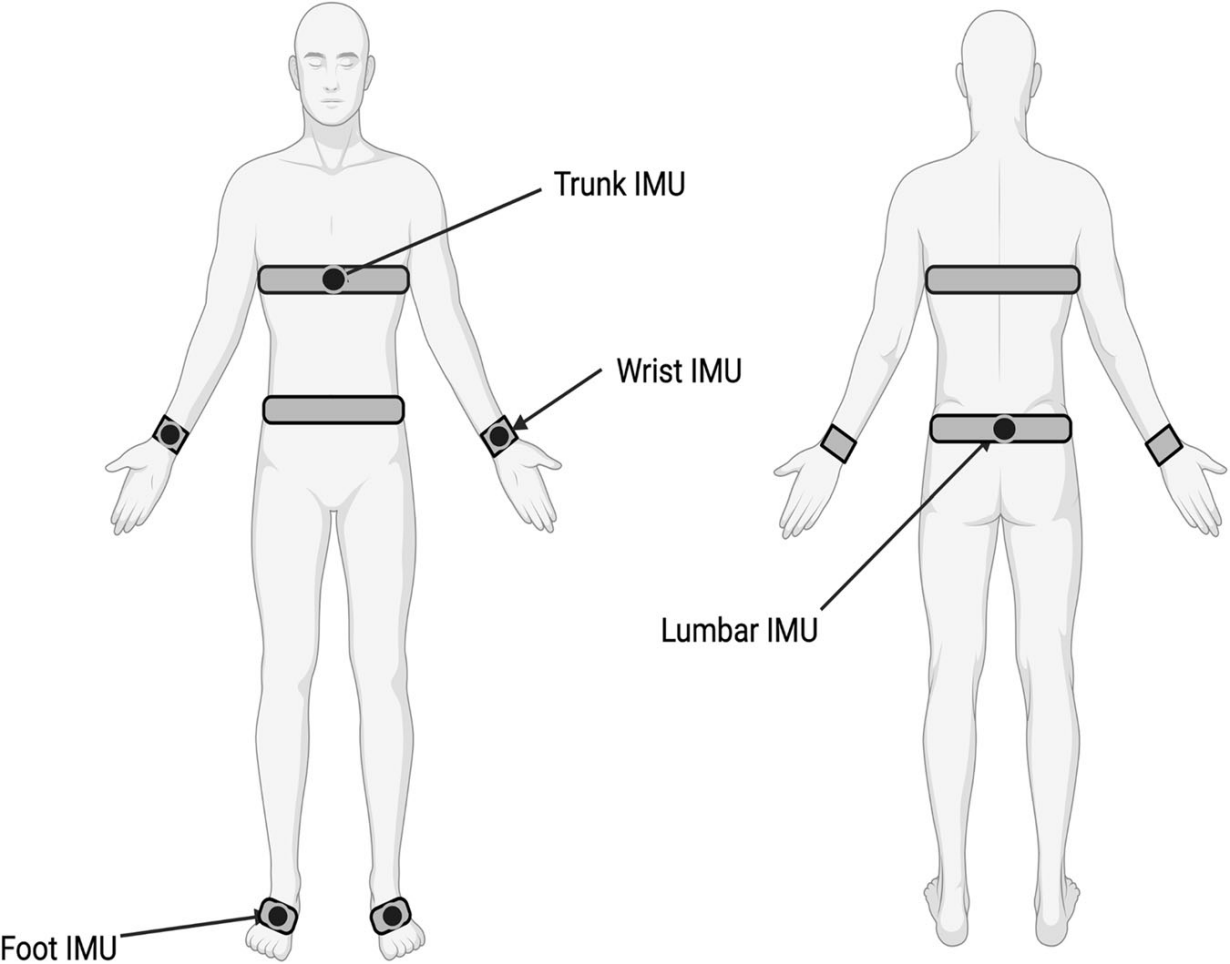
Placement of Inertial Measurement Units (IMUs). Six IMUs were placed on the participants wrists, feet, sternum, and lumbar region.

At their initial visit, participants were asked to walk for two minutes on a straight level surface in a 15 m-long corridor, making 180-degree turns when necessary. For measurement of postural sway, participants were instructed to stand still for 30 s, keeping their eyes closed, with a standard inter-malleolar distance. An experienced clinical researcher was standing or walking on each participant’s side to ensure safety when performing the tasks. An array of 6 inertial measurement unit (IMU) sensors (OpalTM, APDM, Portland, Oregon, USA)^62^ was utilised to collect kinematic data. The sensors were placed on both wrists and feet, the sternum, and the lumbar region, using elastic straps (Fig. 4). Each sensor provided raw accelerometer, gyroscope, and magnetometer data at a sampling frequency of 128 Hz. Clinical history and neurological examination were also obtained. This was done by an MDS-certified researcher using the Movement Disorder Society- Unified Parkinson’s Disease Rating Scale (MDS-UPDRS)^63^, the Hoehn and Yahr scale (H&Y), the Parkinson’s Disease Questionnaire (PDQ39)^64^ and the EQ-5D-5L (UK version)^65^.

### Falls reporting

A fall, similarly to previous studies^66,67^, was defined as an unintentional event that results in a loss of balance and subsequent complete coming to the ground or some lower level, not due to a major intrinsic event or overwhelming hazard.

At each follow-up time point, we recorded simply whether or not the participant had experienced any falls up to that point. Participants in OxQUIP attended study visits every 3 months for 2 years, and on each occasion their clinical history was updated. Falls data for the 24-month time points were obtained from records of these visits. Due to the COVID-19 pandemic, some participants ended the study prior to the 24-month time point; for those participants, telephone follow-up was conducted. At 24 months, 13 participants were followed up via a phone call.

Sixty-month falls follow up data were obtained from two sources. Some participants had continued in research clinics where falls status was recorded. For participants no longer attending research clinics at 60 months (57 participants), telephone follow up was conducted.

### Data reduction and analysis

The MobilityLab software (APDM, Portland, Oregon, USA) was used to automatically extract kinematic features, a list of which can be found in Supplementary Table 1. All features related to stride length were normalised by dividing by the participant’s height in metres. All right and left limb-specific features were relabelled as Ipsilateral and Contralateral, relative to the side of symptom onset.

We aimed to explore the ability of baseline kinematic data to predict the onset of falls at two-time points after the baseline visit, 24 months and 60 months. For each time point, fallers were identified, and the dataset was prepared for analysis.

The collinearities present in the dataset necessitated a feature selection pipeline (Supplementary Fig. 1) for each time point, before applying machine learning techniques. An independent sample comparison of all features was performed using a Mann–Whitney *U* test using the entire cohort of 104 participants, to identify the features that demonstrate the most significant difference between fallers and non-fallers. A 1% false discovery rate (FDR) was used to correct for multiple comparisons.

Next, prior to training the machine learning models, we randomly resampled the majority class (non-fallers) to match the size of the minority class (fallers) at each time point. We selected this under-sampling approach over up-sampling approaches to avoid overfitting^68^. We then trained machine learning models using the identified baseline kinematic features to predict the onset of falls at 24 months and 60 months.

Five machine learning models were then trained to distinguish between fallers and non-fallers at the 24- and 60-month time points: Logistic Regression (LR), Random Forest (RF), Support Vector Machine (SVM), Elastic Net (EN), and XGBoost. The dataset of fallers and non-fallers at each time point was split into training and testing sets using stratified fivefold cross-validation to ensure a balanced representation of fallers and non-fallers in each fold. The full dataset was split into five subsets where four sets were used for training, and the remaining set was used for testing^69^. This training and validation split was performed five times for each instance to be included once in the validation set.

Within each fold, the training and validation sets underwent zero-mean, unit variance standardisation with respect to the train set distribution. Hyperparameter tuning was also performed for each model using Optuna, an automatic hyperparameter optimisation framework. The objective function aimed to maximise the average accuracy across the cross-validation folds. The search space for hyperparameters was defined using Optuna’s distributions (Supplementary Table 5), including categorical, log-uniform, integer, and uniform distributions tailored to each classifier’s specific hyperparameters.

Model performance was evaluated using accuracy, precision, recall, F1 score, and area under the receiver operating characteristic curve (ROC-AUC), averaged across the five cross-validation folds. The ROC curves were generated by averaging the false positive rates (FPR) and true positive rates (TPR) across the cross-validation folds using linear interpolation. 95% confidence intervals for the ROC-AUC were calculated using bootstrapping.

To assess the impact of including baseline clinico-demographic information, age, disease duration, total MDS-UPDRS part III score, and MoCA score were incorporated as additional features. Four distinct feature sets were evaluated for each model and time point:

a) Complete feature set including clinico-demographics

b) Complete feature set excluding clinico-demographics

c) Selected features (based on univariate testing) including clinico-demographics

d) Selected features excluding clinico-demographics

The best-performing models for each feature set and time point were identified based on the average ROC-AUC across the cross-validation folds. The actual and predicted fall status for each participant was recorded for the best models.

## Supplementary information

Supplementary Material

## Data Availability

The datasets used and/or analysed during the current study are available from the corresponding author upon reasonable request.

## Code availability

The underlying code used to analyse this data is available at https://github.com/OxQUIP-Study/FallRiskPD.
