# Supplementary material for: Predicting future fallers in Parkinson’s disease using kinematic data over a period of 5 years
Source: NPJ Digit Med. 2024 Dec 5;7:345. doi: 10.1038/s41746-024-01311-5 (PMC11621420; doi:10.1038/s41746-024-01311-5)
Supplement: Supplementary file 1 — Supplementary Material [file 41746_2024_1311_MOESM1_ESM.pdf]

Supplementary material

<u>Supplementary Table 1</u> – List of features used in the current analysis. Asterisk (*) indicates the features that showed significant difference between fallers and non-fallers.

Lower Limb - Cadence IP (steps/min) [mean]
Lower Limb - Cadence IP (steps/min) [std]
Lower Limb - Cadence CO (steps/min) [mean]
Lower Limb - Cadence CO (steps/min) [std]
Lower Limb - Double Support IP (%GCT) [mean]
Lower Limb - Double Support IP (%GCT) [std]
Lower Limb - Double Support CO (%GCT) [mean]
Lower Limb - Double Support CO (%GCT) [std]
Lower Limb - Elevation at Midswing IP (cm) [mean]
Lower Limb - Elevation at Midswing IP (cm) [std]
Lower Limb - Elevation at Midswing CO (cm) [mean]
Lower Limb - Elevation at Midswing CO (cm) [std]
Lower Limb - Gait Cycle Duration IP (s) [mean]
Lower Limb - Gait Cycle Duration IP (s) [std]
Lower Limb - Gait Cycle Duration CO (s) [mean]
Lower Limb - Gait Cycle Duration CO (s) [std]
Lower Limb - Gait Speed IP (m/s) [mean]
Lower Limb - Gait Speed IP (m/s) [std]
Lower Limb - Gait Speed CO (m/s) [mean]
Lower Limb - Gait Speed CO (m/s) [std]
Lower Limb - Lateral Step Variability IP (cm)
Lower Limb - Lateral Step Variability CO (cm)
Lower Limb - Circumduction IP (cm) [mean]
Lower Limb - Circumduction IP (cm) [std]
Lower Limb - Circumduction CO (cm) [mean]
Lower Limb - Circumduction CO (cm) [std]
Lower Limb - Foot Strike Angle IP (degrees) [mean]
Lower Limb - Foot Strike Angle IP (degrees) [std]
Lower Limb - Foot Strike Angle CO (degrees) [mean]
Lower Limb - Foot Strike Angle CO (degrees) [std]
Lower Limb - Toe Off Angle IP (degrees) [mean]
Lower Limb - Toe Off Angle IP (degrees) [std] *
Lower Limb - Toe Off Angle CO (degrees) [mean]
Lower Limb - Toe Off Angle CO (degrees) [std]
Lower Limb - Single Limb Support IP (%GCT) [mean]
Lower Limb - Single Limb Support IP (%GCT) [std] *
Lower Limb - Single Limb Support CO (%GCT) [mean]
Lower Limb - Single Limb Support CO (%GCT) [std] *
Lower Limb - Stance IP (%GCT) [mean]
Lower Limb - Stance IP (%GCT) [std] *
Lower Limb - Stance CO (%GCT) [mean]
Lower Limb - Stance CO (%GCT) [std] *

Lower Limb - Step Duration IP (s) [mean]
Lower Limb - Step Duration IP (s) [std]
Lower Limb - Step Duration CO (s) [mean]
Lower Limb - Step Duration CO (s) [std] *
Lower Limb - Stride Length IP (m) [mean] *
Lower Limb - Stride Length IP (m) [std]
Lower Limb - Stride Length CO (m) [mean] *
Lower Limb - Stride Length CO (m) [std]
Lower Limb - Swing IP (%GCT) [mean]
Lower Limb - Swing IP (%GCT) [std] *
Lower Limb - Swing CO (%GCT) [mean]
Lower Limb - Swing CO (%GCT) [std] *
Lower Limb - Terminal Double Support IP (%GCT) [mean]
Lower Limb - Terminal Double Support IP (%GCT) [std]
Lower Limb - Terminal Double Support CO (%GCT) [mean]
Lower Limb - Terminal Double Support CO (%GCT) [std] *
Lower Limb - Toe Out Angle IP (degrees) [mean]
Lower Limb - Toe Out Angle IP (degrees) [std]
Lower Limb - Toe Out Angle CO (degrees) [mean]
Lower Limb - Toe Out Angle CO (degrees) [std]
Lumbar - Coronal Range of Motion (degrees) [mean]
Lumbar - Coronal Range of Motion (degrees) [std]
Lumbar - Sagittal Range of Motion (degrees) [mean]
Lumbar - Sagittal Range of Motion (degrees) [std]
Lumbar - Transverse Range of Motion (degrees) [mean]
Lumbar - Transverse Range of Motion (degrees) [std]
Trunk - Coronal Range of Motion (degrees) [mean]
Trunk - Coronal Range of Motion (degrees) [std]
Trunk - Sagittal Range of Motion (degrees) [mean]
Trunk - Sagittal Range of Motion (degrees) [std] *
Trunk - Transverse Range of Motion (degrees) [mean]
Trunk - Transverse Range of Motion (degrees) [std]
Upper Limb - Arm Swing Velocity IP (degrees/s) [mean]
Upper Limb - Arm Swing Velocity IP (degrees/s) [std]
Upper Limb - Arm Swing Velocity CO (degrees/s) [mean]
Upper Limb - Arm Swing Velocity CO (degrees/s) [std]
Upper Limb - Arm Range of Motion IP (degrees) [mean]
Upper Limb - Arm Range of Motion IP (degrees) [std]
Upper Limb - Arm Range of Motion CO (degrees) [mean]
Upper Limb - Arm Range of Motion CO (degrees) [std]
Turn Velocity (degrees/s) [mean]
Turn Velocity (degrees/s) [std]
Steps in Turn (#) [mean]
Steps in Turn (#) [std]
Postural Sway - Acc - 95% Ellipse Axis 1 Radius (m/s^2)
Postural Sway - Acc - 95% Ellipse Axis 2 Radius (m/s^2)
Postural Sway - Acc - 95% Ellipse Rotation (radians)
Postural Sway - Acc - Sway Area (m^2/s^4)
Postural Sway - Acc - Centroidal Frequency (Hz)
Postural Sway - Acc - Centroidal Frequency (Coronal) (Hz)

Postural Sway - Acc - Centroidal Frequency (Sagittal) (Hz)
Postural Sway - Acc - Frequency Dispersion (AD)
Postural Sway - Acc - Frequency Dispersion (Coronal) (AD)
**Postural Sway - Acc - Frequency Dispersion (Sagittal) (AD) ***
Postural Sway - Acc - Jerk (m^2/s^5)
Postural Sway - Acc - Jerk (Coronal) (m^2/s^5)
Postural Sway - Acc - Jerk (Sagittal) (m^2/s^5)
Postural Sway - Acc - Mean Velocity (m/s)
Postural Sway - Acc - Mean Velocity (Coronal) (m/s)
Postural Sway - Acc - Mean Velocity (Sagittal) (m/s)
Postural Sway - Acc - Path Length (m/s^2)
Postural Sway - Acc - Path Length (Coronal) (m/s^2)
Postural Sway - Acc - Path Length (Sagittal) (m/s^2)
Postural Sway - Acc - RMS Sway (m/s^2)
Postural Sway - Acc - RMS Sway (Coronal) (m/s^2)
Postural Sway - Acc - RMS Sway (Sagittal) (m/s^2)
Postural Sway - Acc - Range (m/s^2)
Postural Sway - Acc - Range (Coronal) (m/s^2)
Postural Sway - Acc - Range (Sagittal) (m/s^2)
MDS-UPDRS-III_total
MOCA_total
**Age_years ***
Disease duration

Supplementary Figure 1 Correlation matrix of the selected features for 60 months' time. Collinearities were evident within the selected features list, with strong correlations particularly among the gait variability features. Therefore, the models chosen to predict future fallers were selected because they perform well in the presence of collinearities.

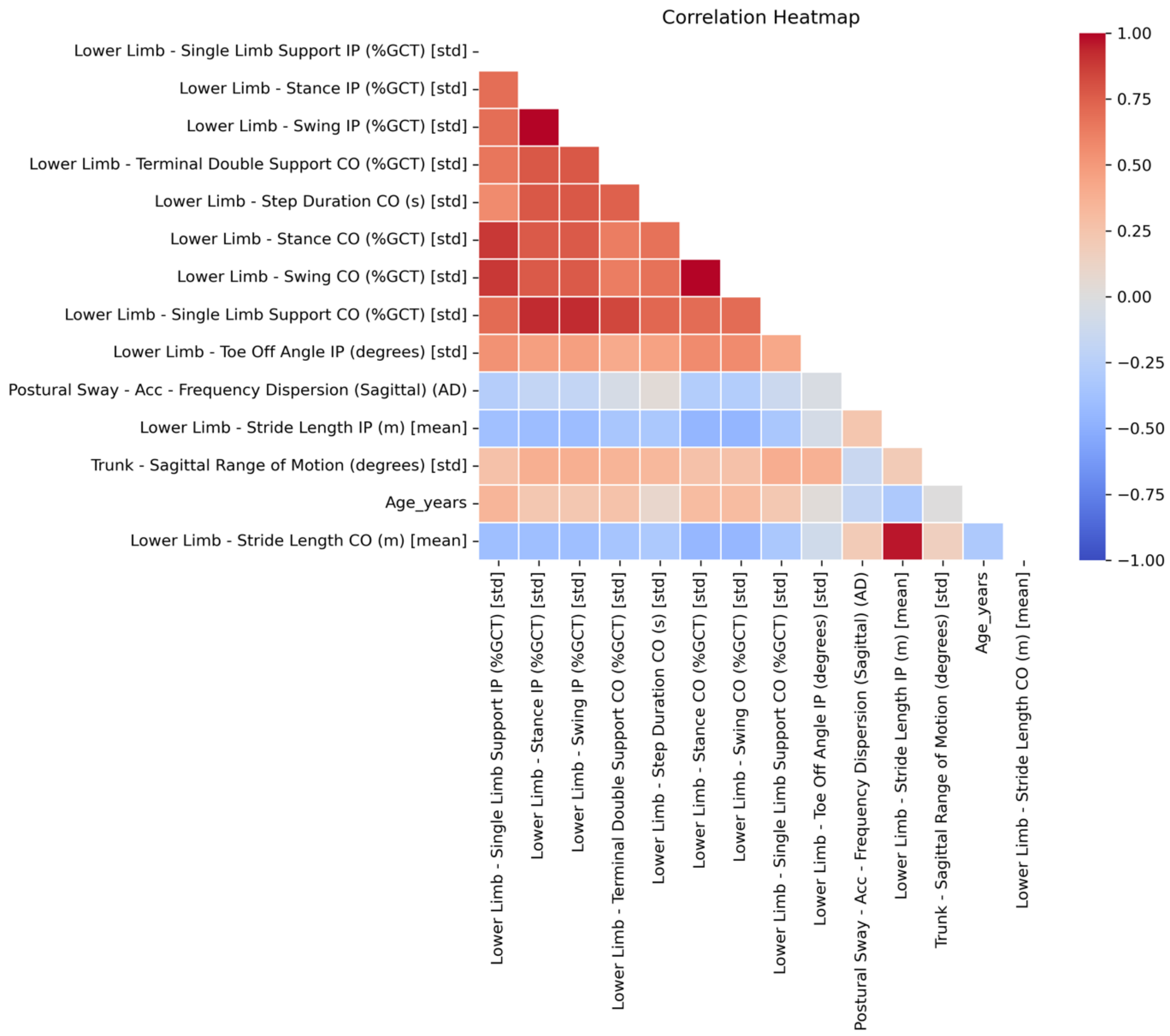

Supplementary Table 2 – Mann-Whitney U statistics for significant features for the 60-month time window

FEATURE	MANN-WHITNEY U	P-VALUE
**LOWER LIMB - TOE OFF ANGLE IP (DEGREES) [STD]**	1140.0	0.0018
**LOWER LIMB - SINGLE LIMB SUPPORT IP (%GCT) [STD]**	1201.5	0.0002
**LOWER LIMB - SINGLE LIMB SUPPORT CO (%GCT) [STD]**	1142.5	0.0017
**LOWER LIMB - STANCE IP (%GCT) [STD]**	1173.5	0.0006
**LOWER LIMB - STANCE CO (%GCT) [STD]**	1148.0	0.0014
**LOWER LIMB - STEP DURATION CO (S) [STD]**	1077.0	0.0012
**LOWER LIMB - STRIDE LENGTH IP (M) [MEAN]**	457.0	0.0024
**LOWER LIMB - STRIDE LENGTH CO (M) [MEAN]**	464.0	0.003
**LOWER LIMB - SWING IP (%GCT) [STD]**	1173.5	0.0006

**LOWER LIMB - SWING CO (%GCT) [STD]**	1148.0	0.0014
**LOWER LIMB - TERMINAL DOUBLE SUPPORT CO (%GCT) [STD]**	1158.5	0.001
**TRUNK - SAGITTAL RANGE OF MOTION (DEGREES) [STD]**	1126.0	0.0028
**POSTURAL SWAY - ACC - FREQUENCY DISPERSION (SAGITTAL)**	454.5	0.0023
**AGE_YEARS**	1124.5	0.0028

Supplementary Table 3 – Model performance evaluation

MODEL	HYPER-PARAMETERS	ACCURACY	PRECISION	RECALL	F1	ROC-AUC	ROC-AUC 95% CI LOWER	ROC-AUC 95% CI UPPER	TIME WINDOW (MONTHS)	FEATURE SET (ALL/SELECTED)	CLINICO-DEMOGRAPHICS INCLUDED (YES/NO)
**LOG_REG**	{'clf__C': 0.0031958012947468166, 'clf__max_iter': 2132, 'clf__solver': 'newton-cg', 'threshold': 0.4557782935917123}	0.92	0.883	1.0	0.931	0.967	0.85	1.0	24	all	Yes
**RF**	{'clf__n_estimators': 1611, 'clf__max_depth': 28, 'clf__min_samples_split': 6, 'clf__min_samples_leaf': 3, 'clf__max_features': 'log2', 'threshold': 0.3896642860695312}	0.84	0.8	1.0	0.876	0.967	0.85	1.0	24	all	Yes
**XGB**	{'clf__max_depth': 20, 'clf__n_estimators': 1163, 'clf__learning_rate': 0.03901758155814582, 'clf__subsample': 0.8087678236908055, 'clf__colsample_bytree': 0.3289839451991656, 'clf__min_child_weight': 1, 'clf__scale_pos_weight': 15.287063329122024, 'thres	0.88	0.883	0.9	0.865	0.9	0.8	1.0	24	all	Yes
**ELASTIC_NET**	{'clf__C': 0.3539925110867631, 'clf__l1_ratio': 0.21101297273980643, 'threshold': 0.2638918207845958}	0.92	0.883	1.0	0.931	0.967	0.85	1.0	24	all	Yes
**SVM**	{'clf__C': 18.417379710276947, 'clf__kernel': 'linear', 'clf__gamma': 2.2066080427722197, 'clf__degree': 2, 'threshold': 0.44441217013110923}	0.887	0.883	0.933	0.891	0.967	0.85	1.0	24	all	Yes
**LOG_REG**	{'clf__C': 0.26897846822404786, 'clf__max_iter': 1303, 'clf__solver': 'sag', 'threshold': 0.20495003515533272}	0.92	0.883	1.0	0.931	0.967	0.85	1.0	24	all	No
**RF**	{'clf__n_estimators': 1194, 'clf__max_depth': 39, 'clf__min_samples_split': 11, 'clf__min_samples_leaf': 6, 'clf__max_features': 'log2', 'threshold': 0.43943314192880295}	0.88	0.883	0.933	0.891	0.967	0.85	1.0	24	all	No
**XGB**	{'clf__max_depth': 65, 'clf__n_estimators': 1884, 'clf__learning_rate': 0.017265755823930758, 'clf__subsample': 0.6041990595039644, 'clf__colsample_bytree': 0.5142043041554412,	0.84	0.833	0.9	0.836	0.867	0.65	1.0	24	all	No

	'clf__min_child_weight': 1, 'clf__scale_pos_weight': 6.407414089018169, 'thres										
**ELASTIC_NET**	{'clf__C': 25.770874102861562, 'clf__l1_ratio': 0.8026110897984811, 'threshold': 0.25392576867921995}	0.92	0.883	1.0	0.931	0.967	0.85	1.0	24	all	No
**SVM**	{'clf__C': 199.80152798543273, 'clf__kernel': 'linear', 'clf__gamma': 0.5911733057714688, 'clf__degree': 2, 'threshold': 0.4353063683428038}	0.92	0.883	1.0	0.931	0.967	0.85	1.0	24	all	No
**LOG_REG**	{'clf__C': 2.3962368855975105, 'clf__max_iter': 1113, 'clf__solver': 'sag', 'threshold': 0.23651968528040954}	0.92	0.883	1.0	0.931	0.967	0.85	1.0	24	selected	Yes
**RF**	{'clf__n_estimators': 1928, 'clf__max_depth': 59, 'clf__min_samples_split': 5, 'clf__min_samples_leaf': 3, 'clf__max_features': 'log2', 'threshold': 0.4515188890070245}	0.84	0.85	0.933	0.865	0.933	0.8	1.0	24	selected	Yes
**XGB**	{'clf__max_depth': 28, 'clf__n_estimators': 396, 'clf__learning_rate': 0.03537805263742179, 'clf__subsample': 0.7287082880755303, 'clf__colsample_bytree': 0.6170373651127488, 'clf__min_child_weight': 1, 'clf__scale_pos_weight': 11.949015906299234, 'thresh	0.92	0.883	1.0	0.931	0.867	0.65	1.0	24	selected	Yes
**ELASTIC_NET**	{'clf__C': 16.149733534459454, 'clf__l1_ratio': 0.19522477318519407, 'threshold': 0.1401421587363022}	0.92	0.883	1.0	0.931	0.967	0.85	1.0	24	selected	Yes
**SVM**	{'clf__C': 0.003422465684088275, 'clf__kernel': 'linear', 'clf__gamma': 0.7059288175689866, 'clf__degree': 3, 'threshold': 0.4137859111229378}	0.92	0.883	1.0	0.931	0.967	0.85	1.0	24	selected	Yes
**LOG_REG**	{'clf__C': 0.9202071986835539, 'clf__max_iter': 1912, 'clf__solver': 'liblinear', 'threshold': 0.26101694468144165}	0.92	0.883	1.0	0.931	0.967	0.85	1.0	24	selected	No
**RF**	{'clf__n_estimators': 1815, 'clf__max_depth': 56, 'clf__min_samples_split': 5, 'clf__min_samples_leaf': 3, 'clf__max_features': 'log2', 'threshold': 0.4604917632766704}	0.88	0.883	0.933	0.891	0.933	0.8	1.0	24	selected	No
**XGB**	{'clf__max_depth': 25, 'clf__n_estimators': 1441, 'clf__learning_rate': 0.0877673357652389, 'clf__subsample': 0.8754395360252616, 'clf__colsample_bytree': 0.5187519517473727, 'clf__min_child_weight': 1, 'clf__scale_pos_weight': 10.16525861519159, 'thresho	0.92	0.883	1.0	0.931	0.867	0.65	1.0	24	selected	No

**ELASTIC_NET**	{'clf__C': 2.5476395643027065, 'clf__l1_ratio': 0.6852508515754069, 'threshold': 0.3440449604112895}	0.92	0.883	1.0	0.931	0.967	0.85	1.0	24	selected	No
**SVM**	{'clf__C': 0.005921083452658545, 'clf__kernel': 'linear', 'clf__gamma': 1.9669215856292483, 'clf__degree': 5, 'threshold': 0.3567451270650225}	0.92	0.883	1.0	0.931	0.967	0.85	1.0	24	selected	No
**LOG_REG**	{'clf__C': 0.005642086188488141, 'clf__max_iter': 714, 'clf__solver': 'sag', 'threshold': 0.545600680242065}	0.74	0.893	0.59	0.678	0.676	0.215	0.978	60	all	Yes
**RF**	{'clf__n_estimators': 1533, 'clf__max_depth': 38, 'clf__min_samples_split': 21, 'clf__min_samples_leaf': 4, 'clf__max_features': 'sqrt', 'threshold': 0.5023481832290014}	0.742	0.808	0.72	0.72	0.778	0.453	0.975	60	all	Yes
**XGB**	{'clf__max_depth': 69, 'clf__n_estimators': 1756, 'clf__learning_rate': 0.0005433709951144718, 'clf__subsample': 0.9905655545211147, 'clf__colsample_bytree': 0.45750649846014624, 'clf__min_child_weight': 1, 'clf__scale_pos_weight': 9.061855669409242, 'thr	0.698	0.741	0.68	0.673	0.71	0.323	0.967	60	all	Yes
**ELASTIC_NET**	{'clf__C': 0.06088388286812541, 'clf__l1_ratio': 0.37512236048430037, 'threshold': 0.5165499764027633}	0.74	0.92	0.58	0.652	0.694	0.227	0.984	60	all	Yes
**SVM**	{'clf__C': 0.001577389532512457, 'clf__kernel': 'poly', 'clf__gamma': 16.718616090057317, 'clf__degree': 3, 'threshold': 0.5226084282511976}	0.607	0.833	0.31	0.432	0.459	0.022	0.919	60	all	Yes
**LOG_REG**	{'clf__C': 0.005393294307290948, 'clf__max_iter': 852, 'clf__solver': 'liblinear', 'threshold': 0.5297318355566222}	0.718	0.86	0.55	0.635	0.666	0.205	0.978	60	all	No
**RF**	{'clf__n_estimators': 1720, 'clf__max_depth': 56, 'clf__min_samples_split': 6, 'clf__min_samples_leaf': 4, 'clf__max_features': 'sqrt', 'threshold': 0.5167365470221826}	0.7	0.764	0.68	0.682	0.738	0.39	0.975	60	all	No
**XGB**	{'clf__max_depth': 40, 'clf__n_estimators': 1523, 'clf__learning_rate': 0.0010866361219808275, 'clf__subsample': 0.9896743893751013, 'clf__colsample_bytree': 0.6835330180368607, 'clf__min_child_weight': 1, 'clf__scale_pos_weight': 3.498467330178081, 'thre	0.718	0.827	0.64	0.666	0.658	0.239	0.975	60	all	No
**ELASTIC_NET**	{'clf__C': 0.042849851622108616, 'clf__l1_ratio': 0.21058763953491505, 'threshold': 0.49941009313963086}	0.696	0.833	0.59	0.624	0.772	0.332	1.0	60	all	No

**SVM**	{'clf__C': 0.030846198418782673, 'clf__kernel': 'poly', 'clf__gamma': 4.998807815398268, 'clf__degree': 4, 'threshold': 0.5506922994372738}	0.567	0.7	0.23	0.313	0.5	0.152	0.867	60	all	No
**LOG_REG**	{'clf__C': 0.7744747828794467, 'clf__max_iter': 2142, 'clf__solver': 'liblinear', 'threshold': 0.5045445296312534}	0.784	0.9	0.71	0.742	0.81	0.41	1.0	60	selected	Yes
**RF**	{'clf__n_estimators': 1858, 'clf__max_depth': 53, 'clf__min_samples_split': 16, 'clf__min_samples_leaf': 3, 'clf__max_features': 'log2', 'threshold': 0.5990544046759362}	0.784	0.96	0.63	0.712	0.848	0.519	1.0	60	selected	Yes
**XGB**	{'clf__max_depth': 28, 'clf__n_estimators': 137, 'clf__learning_rate': 0.00761805803260816, 'clf__subsample': 0.777047964205214, 'clf__colsample_bytree': 0.7429468604292498, 'clf__min_child_weight': 9, 'clf__scale_pos_weight': 15.477691016018344, 'thresho	0.784	0.92	0.68	0.719	0.812	0.479	0.992	60	selected	Yes
**ELASTIC_NET**	{'clf__C': 0.6049813921417699, 'clf__l1_ratio': 0.7428944242438275, 'threshold': 0.4884238159906587}	0.807	0.9	0.75	0.778	0.8	0.388	1.0	60	selected	Yes
**SVM**	{'clf__C': 0.006733533204648455, 'clf__kernel': 'linear', 'clf__gamma': 0.0007107851511125971, 'clf__degree': 5, 'threshold': 0.5192326659196909}	0.807	0.96	0.67	0.76	0.83	0.509	1.0	60	selected	Yes
**LOG_REG**	{'clf__C': 0.7213436763518033, 'clf__max_iter': 923, 'clf__solver': 'sag', 'threshold': 0.5015732300425518}	0.807	0.927	0.71	0.76	0.828	0.452	1.0	60	selected	No
**RF**	{'clf__n_estimators': 1085, 'clf__max_depth': 33, 'clf__min_samples_split': 5, 'clf__min_samples_leaf': 4, 'clf__max_features': 'log2', 'threshold': 0.6121568440899445}	0.784	0.96	0.63	0.712	0.812	0.482	1.0	60	selected	No
**XGB**	{'clf__max_depth': 70, 'clf__n_estimators': 1247, 'clf__learning_rate': 0.0034069563196480945, 'clf__subsample': 0.5264444276434259, 'clf__colsample_bytree': 0.43372511563091226, 'clf__min_child_weight': 2, 'clf__scale_pos_weight': 14.309653925856248, 'th	0.784	0.96	0.63	0.712	0.788	0.428	1.0	60	selected	No
**ELASTIC_NET**	{'clf__C': 7.240237570756752, 'clf__l1_ratio': 0.16377671036581645, 'threshold': 0.6030452811371839}	0.807	0.96	0.66	0.74	0.772	0.432	1.0	60	selected	No
**SVM**	{'clf__C': 0.04781126512504679, 'clf__kernel': 'linear', 'clf__gamma': 0.027506361130383673, 'clf__degree': 2, 'threshold': 0.5305281243732878}	0.807	0.92	0.71	0.773	0.8	0.401	1.0	60	selected	No

Supplementary Table 4 – Confusion Matrix for the 60-month time period. Non-faller cohort (N=74) was resampled to match the sample of fallers (N=23).

True label	non-faller	22	1
	faller	9	14
		non-faller	faller
	Predicted label		

Supplementary Table 5 – Range of Hyperparameters Tuned

MODEL	PARAMETER	RANGE/CHOICES
**LOGISTIC REGRESSION**	C	1e-4 to 1e2
	max_iter	100 to 3000
	solver	['liblinear', 'newton-cg', 'sag']
**RANDOM FOREST**	n_estimators	100 to 2000
	max_depth	10 to 80
	min_samples_split	5 to 50
	min_samples_leaf	2 to 20
	max_features	['sqrt', 'log2', None]
**XGBOOST**	max_depth	20 to 70
	n_estimators	100 to 2000
	learning_rate	1e-4 to 1e-1
	Sub_sample	0.5 to 1.0
	Col_sample_bytree	0.3 to 0.8
	min_child_weight	1 to 20
	scale_pos_weight	1 to 20
**ELASTIC NET**	C	1e-4 to 1e2
	l1_ratio	0.1 to 0.9
**SVM**	C	1e-4 to 1e4
	kernel	['linear', 'rbf', 'poly', 'sigmoid']
	gamma	1e-4 to 1e2
	degree	2 to 5
